# Reconstructing Dermatology Education: The Backward Design and Curricular Content Expansion

**DOI:** 10.1111/tct.70200

**Published:** 2025-09-04

**Authors:** Ellen Overson, Aakanksha Angra

**Affiliations:** ^1^ University of Minnesota Medical School Minneapolis Minnesota USA; ^2^ American Society of Hematology Washington DC USA

## Abstract

**Background:**

Despite the high prevalence of skin conditions, access to dermatologists remains limited, leaving patients to rely on primary care doctors, paediatricians or emergency medicine providers for diagnosis and treatment. Additionally, dermatology education in medical school is often insufficient, with limited hours dedicated to the specialty. The widespread need for dermatologic care and the curricular time devoted to training medical students in dermatology topics are misaligned, which underscores the importance of enhancing dermatology training within the undergraduate medical curriculum.

**Approach:**

To address this gap, we expanded the existing curriculum to create a dermatology course which showcased foundational subjects such as anatomy, physiology, histology, pathology and biochemistry in the context of the body's largest organ: the skin. We collaborated with dermatologists, physicians, scientists and education specialists to systematically design and align all aspects of the course content and assessments with specific learning objectives to ensure practical, real‐world relevance.

**Evaluation:**

The course received positive feedback from students, who appreciated the diverse representation of skin conditions across various skin tones, the case‐based learning experiences, use of in‐person and remote learning formats and the use of practice questions and interactive modules. All students successfully passed the course.

**Implications:**

Curriculum design is an iterative process, and we hope to refine the course based on student feedback and our experiences. We hope that by giving students more time in the curriculum devoted to understanding skin diseases, they will feel more confident diagnosing and managing skin conditions in medical school and beyond.

## 1 Background

Medical schools in the United States have a significant gap in dermatology education. Surveys show significant variation in whether a preclinical dermatology curriculum is required for medical students, and if it is, the content is often less than 10 h [1]. This limited exposure is concerning given that skin conditions are among the most common reasons for medical visits. Analysis of the 2007–2016 National Ambulatory Medical Care Survey found that about half of the visits for the five most common skin diseases were managed by non‐dermatologists [2]. Despite the high burden of skin disease, dermatology is underrepresented on standardised medical exams. The US Medical Licensing Exam (USMLE) Step 1 includes dermatology topics within the musculoskeletal, skin and subcutaneous tissue category, which accounts for only 6%–10% of the exam [3]. Similarly, dermatology comprises just 3% of the internal medicine (IM) board certification exam [4] and 4% of the paediatric board certification exam [5], with no specified content breakdown for the family medicine (FM) board exam.

Surveys show significant variation in whether a preclinical dermatology curriculum is required for medical students, and if it is, the content is often less than 10 h.

The consequences of insufficient dermatology education are evident. In a 2005 survey, only 37% of IM and FM residents felt adequately prepared to diagnose common dermatologic diseases [6]. Among dermatology residents, 77% reported a need for more wound care education, and 70%–80% felt unprepared to manage acute and chronic wounds [7]. Furthermore, clinical trials have shown that dermatology consultation improves diagnostic accuracy and reduces antibiotic use [8, 9]. The disconnect between the limited dermatology training provided in medical school and the high volume of dermatologic conditions seen in clinical practice highlights the urgent need to enhance dermatology education. Strengthening dermatology training in the medical curriculum is a critical first step towards preparing future physicians to provide comprehensive patient‐centred care that aligns with the evolving demands of the healthcare landscape.

The disconnect between the limited dermatology training provided in medical school and the high volume of dermatologic conditions seen in clinical practice highlights the urgent need to enhance dermatology education.

## 2 Approach

Beginning in 2021, we undertook the development of a novel dermatology course as part of a comprehensive shift of the preclinical medical school curriculum from discipline‐centred to system‐based courses. Guidelines, processes, sequence and duration of the courses are governed by the Medical School Education Committee, which oversees all courses. The dermatology course was the second course in the sequence and was allotted 3 weeks. This paper outlines our approach to designing the course (Tables 1, 2, 3) that encompassed the clinical facets of dermatology (e.g., descriptive terminology of skin findings, clinical presentations and natural history of skin diseases, diagnostic and treatment methodologies and skin‐focused patient interview and examination techniques). This curriculum reorganisation was a collaborative effort with a team of 29 subject matter experts (SMEs) composed of physicians and scientists with various specialties and five education specialists.

**TABLE 1:** Week 1: Laying the foundation: The goal of this week is to introduce dermatology terminology and key definitions and to outline skin structures and their functions. Key principles about topical medications are introduced in addition to other commonly used medications in dermatology. Skin disorders are grouped into a few different categories and are gradually introduced, starting with more common, less complex conditions.

Sample session titles	Instruction method	Assessment method
Structure and function of skin Pharmacological principles in dermatology Skin changes across the lifespan + common skin lesions Cosmetic and procedural dermatology Hair and nail disorders	Short pre‐recorded videos	Formative assessment Practice assessment questions within videos or modules or activities Board‐style assessment questions
Pharmacology of pilosebaceous disorders Skin colour and disorders of pigmentation Ultraviolet radiation and skin Research ethics *—An introduction to research ethics was a necessary precursor to discussing skin findings of syphilis (findings that were uncovered using racist and unethical methods*	Short pre‐recorded videos with subsequent in‐person or live + virtual case‐based learning activities	
Immunology and physiology of skin	Modules with slides + practice questions	
Introduction to course Pressure injuries and chronic wounds Pilosebaceous disorders	Live + virtual or live + in‐person interactive session	
Joy, beauty and professionalism	Live + virtual patient panel + small group activity	Reflective writing assignment

**TABLE 2:** Week 2: Expanding knowledge through application. Building on foundational concepts from Week 1, this week focused on applying dermatologic principles to more complex scenarios. Through case studies and interactive activities, students deepened their understanding of skin conditions, learning how to assess, diagnose and manage various dermatologic issues in clinical settings.

Sample session titles	Instruction method	Assessment method
Papulosquamous disorders Dermatitis	Short pre‐recorded videos	Formative assessment Practice assessment questions within videos or modules or activities Board‐style assessment questions Peer review of poster projects
Malignant skin lesions Bacterial infections of the skin Viral infections of the skin	Short pre‐recorded videos with subsequent in‐person or live + virtual case‐based learning activities	
Exanthems + enanthems Fungal skin infection Bites, infestations + tick‐borne illnesses with skin manifestations	Live + virtual or live + in‐person interactive session	
Topical over‐the‐counter (OTC) medication Poster Presentation	Small group research + presentation	
Midterm at end of Week 2		Summative assessment multiple‐choice questions

**TABLE 3:** Week 3: Integrating knowledge for clinical practice. In this final week, students consolidated their learning by applying dermatologic concepts to real‐world clinical cases. Emphasis was placed on synthesising knowledge from previous weeks, refining diagnostic skills and practising patient management strategies through interactive case discussions and hands‐on activities.

Sample session titles	Instruction method	Assessment method
Paediatric dermatology	Short pre‐recorded videos	Formative assessment Practice assessment questions within interactive sessions Board‐style assessment questions
Bullous disorders Urticaria, angioedema + reactive erythemas Vitamin and mineral deficiencies with skin manifestations Purpura	Live, virtual or live interactive session	
Skin signs of systemic disease	Live interactive session with timed‐quiz game	
Small group case‐based learning Facilitators are present but students work through the case as a group, independently conduct research and then return to the group to report out on way they learned in order to come to the appropriate diagnosis and treatment	Student‐directed small group clinical case review	
Small group case reviews	Facilitator‐guided review of multiple clinical vignettes	
Final exam at end of Week 3		Summative assessment with multiple choice questions

Utilising the backward design framework [10], the question that guided our course design was: *What knowledge should every medical student possess upon graduation?* In the process of designing our curriculum, we began by outlining the broad course learning goals. Next, we wrote learning objectives using Bloom's taxonomy model [11], where the action verb of the objective ties to the level of knowledge attainment and cognitive demand to ensure a measurable and multifaceted approach to student learning. To facilitate this process, SMEs from discipline‐specific teams provided input, with the flexibility for the dermatology course directors to provide edits and align with the American Academy of Dermatology (AAD) practice guidelines and recommendations. Key references used at this stage of the curriculum design included *Clinical Dermatology* [12] and Taylor and Kelly's *Dermatology for Skin of Color* [13]. Learning objectives were organised by topic, which guided the session design that went through multiple collaborative iterations.

In our course, we incorporated a variety of session styles (including asynchronous and synchronous sessions) to suit the variety of methods students learn best (Tables 1, 2, 3). Synchronous sessions were either live and in‐person or live and virtual (via online meeting platform). Asynchronous sessions included pre‐recorded videos, e‐learning modules, or article review as prework for a synchronous session. Specifically, e‐learning modules included learning objectives, short segments of didactic content separated by a few formative assessment questions (e.g., multiple‐choice, matching or open‐ended options) to reinforce core concepts. Asynchronous sessions represented about 57% of the course, whereas synchronous sessions made up 43% of the course.

## 3 Evaluation

**FIGURE 1:**
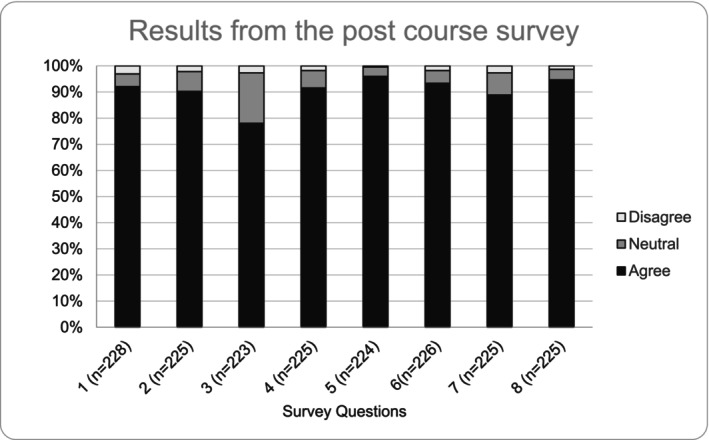
The survey at the end of the course represented in graph format. ‘Agree’ is the combination of ‘strongly agree’ and ‘agree’ responses and ‘disagree’ is the combination of ‘disagree’ and ‘strongly disagree’ responses.

The new dermatology curriculum launched in October 2023 for 244 first‐year allopathic medical students across two campuses. All students successfully passed the course, and 93% completed the post‐course survey (Table 4). Eighty‐nine per cent of the students answered positively that the course content and structure helped them achieve the course learning objectives, and 95% found the course to be valuable. The one category that performed poorly was the recommended resources useful for learning the course material, which received 78% satisfaction. All other questions received a 90% or greater satisfaction rating (Figure 1).

**TABLE 4:** This survey was provided to all students at the end of the course and this table summarises their responses.

Survey questions	Strongly agree	Agree	Neutral	Disagree	Strongly disagree
1 The course learning goal and objectives were clearly communicated (*n* = 228)	107 (47%)	103 (45%)	11 (5%)	6 (3%)	1 (0%)
2 The content taught in the course was aligned with the learning goals and objectives (*n* = 225)	108 (48%)	95 (42%)	17 (8%)	4 (2%)	1 (0%)
3 The recommended resources were useful for learning the course material (*n* = 223)	87 (39%)	87 (39%)	43 (19%)	6 (3%)	0 (0%)
4 The graded assessment(s) appropriately tested the course learning goals and objectives (*n* = 225)	120 (53%)	86 (38%)	15 (7%)	4 (2%)	0 (0%)
5 The course successfully integrated basic science knowledge and clinical practice (*n* = 224)	119 (53%)	96 (43%)	8 (4%)	1 (0%)	0 (0%)
6 The course successfully integrated diversity, equity and inclusion topics (*n* = 226)	118 (52%)	93 (41%)	11 (5%)	3 (1%)	1 (0%)
7 Overall, the course content and structure successfully helped me achieve the course learning goals and objectives (*n* = 225)	108 (48%)	92 (41%)	19 (8%)	3 (1%)	3 (1%)
8 Overall, I found this course to be valuable (*n* = 225)	125 (56%)	88 (39%)	9 (4%)	3 (1%)	0 (0%)
Survey question	Far too much virtual	A little too much virtual	Well‐balanced	A little too much in‐person	Far too much in‐person
How balanced was the course material between the amount of time spent asynchronously/virtually and in‐person? (*n* = 221)	6 (3%)	2 (1%)	136 (62%)	63 (29%)	14 (6%)

As shown in Table 4, 136 students (62% of respondents) found the course to have a good balance between asynchronous and synchronous content, and 85 students (38% of respondents) thought the balance could be improved. Of those who sought improvement, the majority [77 out of 85 (90%)] thought there was too much in‐person content, and only a minority [8 out of 85 (10%)] thought there was too much online content.

Overall, students found the course to be enjoyable. On the post‐course survey, students were asked: ‘what did you find to be the most valuable aspect of the course?’, and their responses were analysed for mention of assignment types. Among responses that referenced assignment types, 56% appreciated the e‐learning modules most, 27% liked the case‐based learning sessions most, and 8% favoured the quiz game session and practice questions during in‐person interactive sessions. When analysing responses to the question ‘do you have suggestions on how we could improve the course?’, responses indicated that preparing for the poster presentation session and completing the e‐learning modules took more time than expected. Students commented that blocking off more time on the calendar for this work would be helpful.

## 4 Implications

Curriculum design is an iterative process that offers opportunities for refinement based on student feedback, instructor experiences and the requirements set forth by the medical school and the Liaison Committee on Medical Education. Whereas many medical schools are making positive curricular changes [14], none have described our use of backward design in this way. Similarly, although the AAD and others [1] have recognised the need for more dermatology training and have made supplemental dermatology training materials, we have not seen descriptions in the literature where dermatology curricular content expansion is implemented and required for all medical students. Further, although our course formation occurred in the context of a comprehensive curricular transformation of all the medical school courses, a comprehensive curricular transformation is not required to expand dermatology education.

Although we are pleased with the success of our first course iteration, we are also excited to explore opportunities for improvement. Regarding the balance between asynchronous and synchronous content, most students felt the course was well balanced. Of the group who sought improvement, most preferred increased asynchronous content. This balance could be adjusted for the future so that foundational concepts in Week 1 are more often asynchronous sessions, whereas more complex diseases discussed in Week 3 may be more appropriately reinforced with in‐person sessions. This would allow for self‐pacing on asynchronous content, yet for more complex material, an in‐person, case‐based design could allow students to apply their knowledge in clinical contexts and receive immediate feedback from expert instructors.

Another opportunity for improvement relates to multi‐system diseases. As the curriculum was being created in a stepwise fashion, it was not always clear whether a future course was planning on covering the same content and to what extent. Lack of discussion between course directors was a recipe for gaps or redundancy in the curriculum. A future step would be to create a system of topic tracking across the entire preclinical curriculum to allow better visibility for content creators and for students.

Another direction we hope to pursue is regarding the assessment of medical student knowledge gains. At this stage, our outcomes mostly represent student reactions to the course. Although we did tie session learning objectives to each of our assessment questions, multiple‐choice questions still have limitations when measuring learning. However, if we utilise the Kirkpatrick model [15] as a framework to identify the impact of the course, we know that reactions are an important part of influencing whether a programme is successful. In the future, we would like to utilise pre‐ and post‐tests to measure knowledge gains and to inform session design. We also hope to analyse more durable knowledge gains for students as they progress through medical school, such as the review of standardised exam performance data, if able, to see how the course redesign may have had an impact.

Ultimately, this curriculum redesign marks a positive step towards increasing the number of hours devoted to dermatology education. However, continued reflection and adaptation are necessary to ensure the course remains relevant and effective. As we gather further feedback from students and faculty, and as we respond to ongoing changes in medical practice and education, future iterations of the course will continue to strengthen its alignment with the needs of both learners and the healthcare system.

This curriculum redesign marks a positive step towards increasing the number of hours devoted to dermatology education.

## Author Contributions

**Ellen Overson:** conceptualization; data curation; formal analysis; writing – original draft; writing – review and editing; supervision. **Aakanksha Angra:** project administration; methodology; writing – review and editing; writing – original draft; formal analysis; data curation; conceptualization.

## Ethics Statement

The University of Minnesota Institutional Review Board (IRB) determined this project (STUDY00022852) was not human research.

## Conflicts of Interest

The authors declare no conflicts of interest.

## Data Availability

The data that support the findings of this study are available on request from the corresponding author. The data are not publicly available due to privacy or ethical restrictions.
